# Inertial Sensor Self-Calibration in a Visually-Aided Navigation Approach for a Micro-AUV

**DOI:** 10.3390/s150101825

**Published:** 2015-01-16

**Authors:** Francisco Bonin-Font, Miquel Massot-Campos, Pep Lluis Negre-Carrasco, Gabriel Oliver-Codina, Joan P. Beltran

**Affiliations:** 1 Systems, Robotics and Vision, Department of Mathematics and Computer Science, University of the Balearic Islands, Cra de Valldemossa, km 7.5, Palma de Mallorca 07122, Spain; E-Mails: miquel.massot@uib.cat (M.M.-C.); pl.negre@uib.cat (P.L.N.-C.); goliver@uib.es (G.O.-C.); 2 Balearic Islands Coastal Observing and Forecasting System (SOCIB), Data Center Parc Bit, Naorte, Bloc A, 2^*O*^p. pta. 3, Palma de Mallorca 07121, Spain; E-Mail: jbeltran@socib.es

**Keywords:** sensor fusion, visual localization, autonomous underwater vehicles, underwater landscape

## Abstract

This paper presents a new solution for underwater observation, image recording, mapping and 3D reconstruction in shallow waters. The platform, designed as a research and testing tool, is based on a small underwater robot equipped with a MEMS-based IMU, two stereo cameras and a pressure sensor. The data given by the sensors are fused, adjusted and corrected in a multiplicative error state Kalman filter (MESKF), which returns a single vector with the pose and twist of the vehicle and the biases of the inertial sensors (the accelerometer and the gyroscope). The inclusion of these biases in the state vector permits their self-calibration and stabilization, improving the estimates of the robot orientation. Experiments in controlled underwater scenarios and in the sea have demonstrated a satisfactory performance and the capacity of the vehicle to operate in real environments and in real time.

## 1. Introduction and Related Work

In the last decade, technology applied to autonomous underwater vehicles (AUVs) has experienced a spectacular development. Scientific, industrial or security applications related to remote observation, object identification, wreck retrieval, mapping and rescuing, among others, have boosted such technological advances. AUVs are progressively becoming fundamental tools for underwater missions, minimizing human intervention in critical situations and automatizing as many procedures as possible. Despite this increasing interest and the great number of vehicles developed far and wide, their cost, size, weight and depth rate are the main factors that mostly restrict their commercialization and extensive use worldwide.

Compared to their larger counterparts, micro-AUVs are smaller, lighter and cheaper to acquire and maintain. These reasons make small AUVs a valuable tool for exploring and mapping shallow waters or cluttered aquatic environments, addressing a wide variety of applications. Conversely, the reduced size of these vehicles limits seriously the equipment that they can carry; thus, they have to be endowed with a reduced sensor set, which can meet, simultaneously, navigation and mission purposes.

The literature is very scarce in this kind of compact system. Smith *et al.* [1] proposed a micro-AUV to perform inspection, hydrographic surveying and object identification in shallow waters. Watson *et al.* [2] detail the design of a micro-AUV specially designed to work in swarms, forming mobile underwater sensor networks for monitoring nuclear storage ponds. The hull of this micro-AUV consists of a hemisphere of 150 mm in diameter, and it contains several mini acoustic sensors, a pressure sensor, a gyroscope and a magnetic compass. Wick *et al.* presented in [3] a miniature AUV for minefield mapping. This robot can work alone or in groups to perform cooperative activities. The dimensions of this vehicle are 60 cm in length by 9 cm in diameter, and it is equipped with a compass, a gyroscope, an accelerometer and a pressure sensor. Both vehicles ([2,3]) have a limited data storage capacity and are mostly prepared to transmit the collected information to an external unit regularly.

Some micro-AUVs incorporate cameras inside their hull, but to the best of our knowledge, none of them have been endowed with two stereo rigs. For instance, Heriot Watt University designed mAUVe [4], a prototype micro-AUV that weighs only 3.5 kg and is equipped with an inertial unit, a pressure sensor and a camera. There are mini-AUVs currently operative that incorporate vision as an important sensor, but all of them have higher dimensions and weight (for instance, DAGON [5], REMUS [6] or SPARUS-II [7]).

In micro-AUVs, navigation, guidance and control modules are usually approached from the point of view of hydrodynamic and kinematic models, since the power of the thrusters is limited by their small volume and light weight, and they are easily influenced by waves or currents [8,9]. These models generate complex non-linear equation systems that usually depend on the structure of the vehicle (mass and shape) and on the external forces that act on it (gravity, buoyancy, hydrodynamics, thrusters and fins) [10]. As a consequence, these models have to be reconsidered if any significant modification is made to the vehicle, adding a considerable complexity to the system without producing, sometimes, the expected results [11]. Furthermore, a control system optimization with respect to its propelling infrastructure and its hydrodynamic behavior is unavoidable, especially if the energy storage and the power supply are limited [12]. An inefficient control strategy will have negative effects on the vehicle autonomy. On the contrary, AUV navigation can be performed by using the data provided by proprioceptive or exteroceptive sensors in an adaptive control unit. This unit estimates continuously the state of the vehicle (pose and twist) regardless of its shape, size and the affecting set of external forces. Assuming general motion models and using inertial measurements to infer the dynamics of the vehicle simplifies the system, facilitates its application to different robots and makes it dependable on the sensor suit rather than on the robot itself [13]. A remarkable approach for cooperative localization of a swarm of underwater robots with respect to a GPS-based geo-referenced surface vehicle is detailed in [14,15]. The algorithm applies a geometric method based on a tetrahedral configuration, and all AUVs are assumed to be equipped with low cost inertial measurement units (IMU), a compass and a depth sensor, but only one of them is equipped with a Doppler velocity log (DVL).

The size and price of equipment have to be optimized when designing micro-AUVs. Low-cost sensors generally provide poor quality measures, so significant errors and drifts in position and orientation can compromise the mission achievement. In this case, software algorithms to infer accurately the vehicle pose and twist become a critical issue since small errors in orientation can cause important drifts in position after a certain time.

Although the vehicle global pose could be inferred by integrating the data provided by an inertial navigation system (INS), in practice, and due to their considerable drift, this information is only valid to estimate motion during a short time interval. Thus, INSs are usually assisted by a suite of other exteroceptive sensors with varying sampling frequencies and resolutions.

A complementary filter [16] could contribute to smoothing the orientation data given by the gyro and accelerometer, and the rest of the position/velocity variables could be taken from the aiding raw sensorial data. However, merging the data of multiple navigation sensors in an extended Kalman filter (EKF) goes one step beyond, since they take into account the motion (dynamic) model of the system, they correct the inertial estimates with external measurements and they consider the uncertainties of all of these variables. Furthermore, they are a widely-extended solution in autonomous robots [17]. Strategies based on Kalman filters also permit the continuous control of the vehicle despite the different frequencies at which the diverse sensors provide measurements and although, for example, any of those sensors may get temporally lost.

The error state formulation of Kalman filters (ESKFs), also called indirect Kalman filters, deal separately with nominal variables and their errors. Although the classical EKF formulation and an ESKF should lead to similar results, the later generates lower state covariance values, and variations on the error variables are much slower than changes on the nominal navigation data [18,19], being better represented as linear. Consequently, ESKFs are especially preferable, but not limited, for vehicles with six degrees of freedom (DOF) with fast dynamics, providing high stability in the navigation performance [20,21]. Besides, ESKF formulations permit continuous vehicle pose estimation, integrating the INS data, in case either the filter or all of the aiding sensors fail.

This paper offers, as a main contribution, a detailed explanation of the structure, the sensorial equipment and the main navigation software components of Fugu-C, a new micro-AUV developed by Systems, Robotics and Vision, a research group of the University of the Balearic Islands (UIB). Fugu-C was initially developed as a testing platform for research developments, but it rapidly became a truly useful tool for underwater image recording, observation, environment 3D reconstruction and mosaicking in shallow waters and in aquatic environments of limited space.

The goal was to implement a vehicle fulfilling a set of key factors and requirements that differentiated it from other micro-AUVs that incorporated some of the same facilities. Fugu-C has the advantage of a micro-AUV in size and weight, but with additional strengths typical of bigger vehicles.

Its most important features are:

(1) The navigation and guidance modules are based uniquely on the sensor suite. The vehicle is equipped with two stereo rigs, one IMU and a pressure sensor. This paper pays special attention to the navigation filter used in Fugu-C to fuse all of the data coming from all of the navigation sensors. The filter is a particularization of an ESKF approach and includes some strategic points present in similar solutions described in [21–24]. Similarly to [21], the state vector includes not only the pose and velocity of the vehicle, but also the biases of the inertial sensors, permitting a rapid compensation of these systematic errors accumulated in the estimates of the vehicle orientation. As for all of these aforementioned pieces of work, this is a multiplicative filter, which means that all orientations are expressed in the quaternion space, avoiding errors due to attitude singularities. Contrary to other similar references, this filter has a general design prepared to work with vehicles with slow or fast dynamics (AUVs or unmanned aerial vehicles (UAV), respectively). Similarly to [23], the error state is reset after each filter iteration, avoiding the propagation of two state vectors and limiting the covariance variations to the error dynamics. While the other referenced ESKF-based solutions assume non-linear and sometimes complex observation models, we propose to simplify the definition of the predicted measurement errors in the Kalman update stage, applying a linearized observation function, which returns the same values contained in the error state vector. Experimental results shown in Section 5 validate our proposal. Furthermore, the implementation in C++ and its wrapper in the robot operating system (ROS, [25]) are published in a public repository for its use and evaluation [26].
(2) Two stereo cameras point to orthogonal directions, covering wider portions of the environment simultaneously. AQUASENSOR [27] is a very similar video structure for underwater imaging. It can be attached to an underwater vehicle, but contrary to Fugu-C, it has only one stereo rig connected to the same hardware structure. The spatial configuration of both cameras is very important to reconstruct, in 3D, the larger parts of the environment with less exploration time and, thus, with less effort. Furthermore, while the down-looking camera can be used for navigation and 3D reconstruction, the frontal one can be used for obstacle detection and avoidance.
(3) Fugu-C can be operated remotely or navigate autonomously describing lawn-mowingpaths at a constant depth or altitude. The autonomy of the vehicle is variable, depending on the capacity of the internal hard-drive and the powering battery.
(4) The software computation capacity and its internal memory outperform the other cited micro-AUVs, which mostly collect data and send them to external computers to be processed.

This work is organized as follows: Section 2 describes the Fugu-C hardware and software platforms. Section 3 details the whole visual framework and functionalities. Section 4 outlines the modular navigation architecture and the detailed design of the error state Kalman filter. Section 5 presents experimental results concerning navigation and 3D reconstruction, and finally, Section 6 extracts some conclusions about the presented work and points out some details concerning forthcoming work.

## 2. Platform Description

### 2.1. Hardware

The Fugu-C structure consists of a cylindrical, transparent, sealed housing containing all of the hardware and four propellers, two horizontal and two vertical. The dimensions of the cylinder are ø212 × 209 mm, and the motor supports are 120 mm long. Despite the fact that the propeller configuration could provide the vehicle with four DOFs, surge, heave, roll and yaw, due to the vehicle dynamics, it is almost passively stable in roll and pitch. Consequently, the robot has three practical DOFs, surge, heave and yaw: (*x*, *z*, *yaw*).

**Figure 1.**
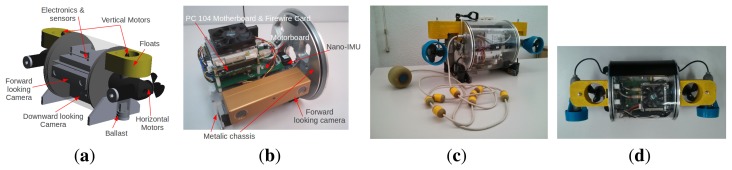
Fugu-C. (**a**) The CAD model of the vehicle; (**b**) the hardware and structure; (**c**,**d**) two different views of the robot.

Figure 1a shows the 3D CAD model design of the vehicle, Figure 1b shows the hardware and the supporting structure, Figure 1c,d show two views of the final result.

**Figure 2.**
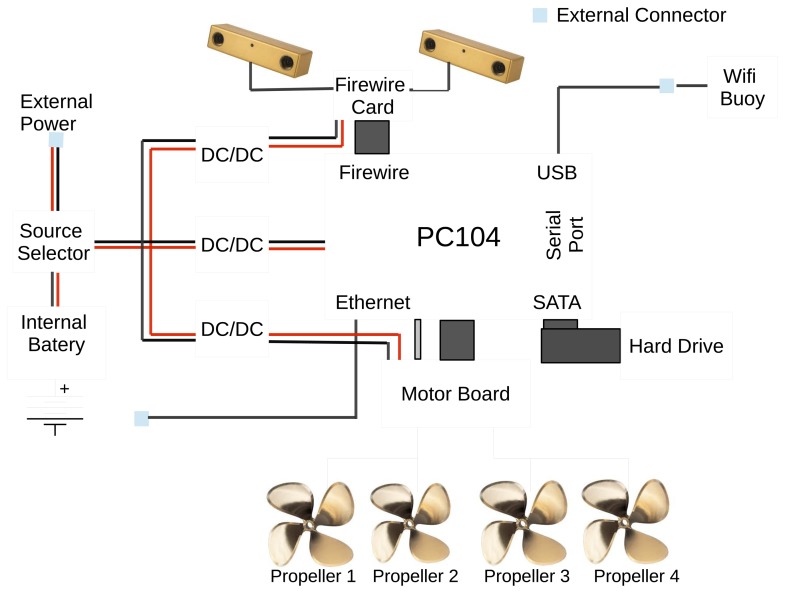
The schematic of the Fugu-C hardware connection.

Fugu-C can operate as an AUV, but it can also be teleoperated as a remotely operated vehicle (ROV) with a low-cost joystick. Its hardware includes (see Figure 2):

- Two IEEE-1394a stereo cameras, one of them oriented toward the bottom, whose lenses provide a 97° horizontal field of view (HFOV), and another one oriented toward the forward direction with a 66° HFOV.
- A 95-Wh polymer Li-Ion battery pack. With this battery, Fugu-C has an autonomy for about 3 h, depending on how much processing is done during the mission. For an autonomous mission with self-localization, the autonomy is reduced to two hours.
- A PC104 board based on an Atom N450 at 1.66 GHz with a 128-GB SSD 2.5-inch hard drive.
- A three port 800 FireWire PC104 card to connect the cameras to the main computer board.
- A nano IMU from Memsense, which provides triaxial acceleration, angular rate (gyro) and magnetic field data [28].
- A power supply management card, which permits turning on/off the system, charging the internal battery and the selection between the external power supply or the internal battery.
- A microcontroller-based serial motor board that manages some water leak detectors, a pressure sensor and four DC motor drivers.
- An external buoy containing a USB WiFi antenna for wireless access from external computers.
- A set of DC-DC converters to supply independent power to the different robot modules.
- Four geared, brushed DC motors with 1170 rpm of nominal speed and 0.54 kg·cm torque.

A 16-pin watertight socket provides the connectivity for wired Ethernet, external power, battery changing and a WiFi buoy. Note that not all connections are needed during an experiment. If the wired Ethernet is used (tethered ROV in use), then the buoy is not connected. Alternatively, if the buoy is connected (wireless ROV/AUV), then the wired Ethernet remains disconnected.

The autonomy of the robot when operating in AUV mode with the current type of battery has been calculated taking into account that its average power consumption at maximum processing (all sensors on, including both cameras, and all of the electronics and drivers operative) and with the motors engaged is around 44.5 W, so the battery would last (96 W·h)/(44.5 W) = 2.15 h at full charge. If it was necessary to include two LED bulbs (10 W) attached to the vehicle enclosure to operate in environments with poor illumination conditions, the internal power consumption would increase to 64.5 W and the battery would last (96 W·h)/(64.5 W) = 1.49 h.

The robot can operate in shallow waters up to 10 m in depth. However, another version for deeper waters could be made by thickening the acrylic cylinder.

Experiments showed an average speed in surge of 0.5 m/s and an average speed in heave of 1 m/s.

### 2.2. Software

All of the software was developed and run using ROS middleware [25] under GNU/Linux. Using ROS makes the software integration and interaction easier, improves their maintainability and sharing and strengthens the data integrity of the whole system. The ROS packages installed in Fugu-C can be classified into three categories: internal state management functions, vision functions and navigation functions.

The internal state management function continuously checks the state of the water leak sensors, the temperature sensor and the power level. The activation of any of these three alarms induces the system to publish a ROS topic-type alarm, to terminate all running software and to shutdown the horizontal motors, while it activates the vertical ones to launch the vehicle quickly up to the surface.

The visual functions include image grabbing, stereo odometry, 3D point cloud computation and environment reconstruction, which is explained in Section 3.

The navigation functions include the MESKF for sensor fusion and pose estimation and all of the modules for twist and depth control, and it is detailed in Section 4.

## 3. The Vision Framework

### 3.1. Image Grabbing and Processing

In order to save transmission bandwidth, raw images provided by any of the stereo cameras are encoded using a Bayer filter and sent directly to the computer, where they are processed as follows:

(1) First, RGB and gray-scale stereo images are recovered by applying a *debayer* interpolation.
(2) Then, these images are rectified to compensate for the stereo camera misalignment and the lens and cover distortion; the intrinsic and extrinsic camera parameters for the rectification are obtained in a previous calibration process.
(3) Optionally, they are downscaled from the original resolution (1024 × 768) to a parameterizable lower rate (power of two divisions); this downsampling is done only if there is a need to monitor the images from remote computers without saturating the Ethernet communications. Normally, a compressed 512 × 384 px image is enough to pilot the vehicle as an ROV without compromising the network capacity. If the vehicle is being operated autonomously, this last step is not performed.
(4) Following the rectification, disparity calculation is done using OpenCV's block matching algorithm to finally project these disparity images as 3D points.
(5) Odometry is calculated from the bottom-looking camera with the LibViso2 library. In our experiments, the forward-looking camera has been used only for monitoring and obstacle detection, although it could also be used to compute the frontal 3D structure.

**Figure 3.**
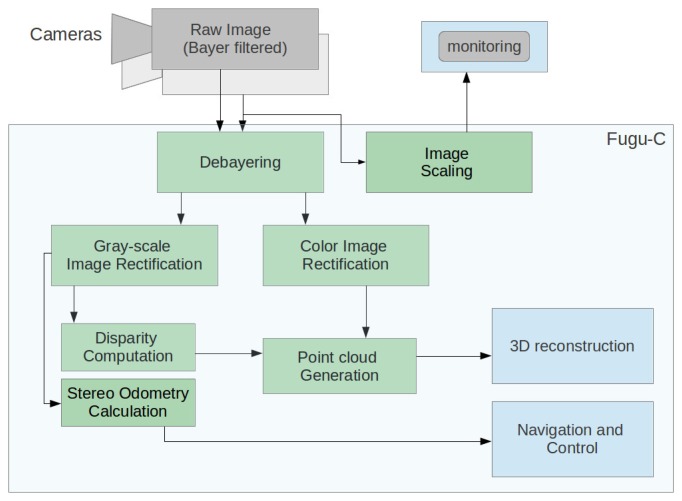
The successive steps of the on-line image processing task.

Figure 3 shows the image processing task sequence performed in Fugu-C.

### 3.2. Visual Odometry

Stereo visual odometry is used to estimate the displacement of Fugu-C and its orientation. Many sophisticated visual odometry approaches can be found in the literature [29]. An appropriate approach for real-time applications that uses a relatively high image frame rate is the one provided by the Library for Visual Odometry 2 (LibViso2) [30]. This approach has shown good performance and limited drift in translation and rotation, in controlled and in real underwater scenarios [31].

The main advantages derived from LibViso2 and that make the algorithm suitable for real-time underwater applications are three-fold: (1) it simplifies the feature detection and tracking process, accelerating the overall procedure; in our system, the odometer can be run at 10 fps using images with a resolution of 1024 × 768 pixels; (2) a pure stereo process is used for motion estimation, facilitating its integration and/or reutilization with the module that implements the 3D reconstruction; (3) the great number of feature matches found by the library at each consecutive stereo image makes it possible to deal with high resolution images, which is an advantage to increase the reliability of the motion estimates.

### 3.3. Point Clouds and 3D Reconstruction

3D models of natural sea-floor or underwater installations with strong relief are a very important source of information for scientists and engineers. Visual photo-mosaics are a highly valuable tool for building those models with a high degree of reliability [32]. However, they need a lot of time to be obtained, and they are rarely applicable online. On the contrary, the concatenation of successive point clouds registered by accurate pose estimates permits building and watching the environment online and in 3D, but with a lower level of detail. Thus, photo-mosaicking and point cloud concatenation can be viewed at present as complementary instruments for optic-based underwater exploration and study.

Dense stereo point clouds computed from the disparity maps [33] are provided by Fugu-C at the same rate as the image grabber. They are accumulated to build a dense 3D model of the underwater environment, visible online if a high rate connection between the vehicle and an external computer is established. The concatenation and meshing of the partial reconstructed regions must be done according to the robot pose. Although the odometric position (*x*, *y*, *z*) accumulates drift during relatively long routes, the system can reconstruct, with a high degree of reliability, static objects or short-medium stereo sequences, if no important errors in orientation are accumulated. However, if the orientation of the vehicle diverges from the ground truth, especially in roll and pitch, the successive point clouds will be concatenated with evident misalignments, presenting differences in inclination between them, thus distorting the final 3D result.

In order to increase the reliability of the reconstructed areas in longer trajectories and to minimize the effect of the orientation errors in the 3D models, we have implemented a generic ESKF, which corrects the vehicle attitude estimated by the visual odometer in the three rotation axis and minimizes the effect of the gyroscope drift.

## 4. Navigation and Localization

### 4.1. Architecture Pipeline

The navigation architecture of Fugu-C is designed at three concatenated levels: (1) a twist controller; (2) a depth controller; and (3) a motion planner.

The twist control module is, in turn, implemented in three interdependent layers, namely:

- Motor layer: the orders directly sent to the motor board node, in charge of interacting with the four propellers.
- Wrench layer: translational (thrust) and angular (torque) forces calculated to move the vehicle in a desired direction with a certain speed are the inputs (wrench levels) of the thruster allocation module. Wrench levels are transformed into motor levels according to the affine thruster model and through a thruster allocation matrix (TAM) [34].
- Twist layer: twist includes the linear and angular velocities. The inputs of this module are the velocity setpoint and the current velocity. This module performs a basic proportional integral derivative (PID) controller for each degree of freedom (x, y, z; roll, pitch, yaw) which outputs a wrench level, containing the necessary force and torque to reach the requested twist.

During the experiments with Fugu-C in the pool and in the sea, the PID gains were experimentally adjusted in order to obtain an adequate response of the vehicle in each scenario, avoiding instabilities, oscillations or steady-state errors. A systematic calculation of the PID gains is included in an upcoming work, but excluded in the scope of this paper.

The depth module is also a basic PID that outputs a wrench level in the *z*axis from two inputs: the current depth and the depth setpoint. The PID gains of these two additional modules are also tuned experimentally and online during each test.

The motion planner module is in charge of generating the twist set points for the twist controller. It can be either a joystick to control the vehicle remotely or a software module that generates automatically the needed twist, depending on the location of the goal point and the current vehicle state in terms of pose and velocity.

### 4.2. Sensor Fusion Using an Indirect Kalman Filter

#### 4.2.1. Overview

Measurements of inertial sensors always include a certain systematic error, the so-called bias. For example, in the case of gyros, bias is the return of the sensor when the body is rotationless. The desirable situation would be to expect a fixed bias, but unfortunately, biases tend to vary, for instance, with temperature, voltage, due to noise and over time (in gyros, the time varying error is commonly known as drift). The accumulated drift, sample after sample, generates an important deviation of the final pose estimation integrated from the INS measurements with respect to the ground truth. Some manufacturers give an estimation of these systematic errors, assuming they are constant, but in practice, they are not. Consequently, it turns out to be very difficult to compensate for their effect. The proposed navigation filter includes, in its state vector, the systematic errors of the inertial sensors, assuming, initially, that those values will be constant. The results will show how their stabilized values are bounded and small, permitting their easy compensation. The effects of this compensation are especially evident in the estimation of the vehicle orientation, as shown later in Section 5.

Representing the vehicle orientation in the quaternion space is essential in vehicles with six DOF to prevent singularities in the attitude estimation [35]. However, the quaternion 4 × 4 covariance matrix has rank three, due to the unit norm constraint, and, thus, can present singularities. One alternative to solve this problem is to represent the vehicle attitude as a quaternion and the attitude error as a three-component rotation vector [23].

The data coming from the pressure sensor, the IMU and the camera were integrated in a multiplicative (quaternion-based) error state Kalman filter (MESKF). The pose and twist outputted by this filter were, in turn, used as inputs for the motion planner and for the twist controller described above. Although Fugu-C has, at the moment, only three DOF (surge, heave and yaw), the MESKF has a generic design, permitting the attachment of as many sensors as needed and ready to work in vehicles with up to six DOF. A brief outline of this filter was firstly presented by some of the same authors in [36]. Here, an extended and detailed description is offered to the reader, while the results of its application in Fugu-C are shown in Section 5.2.

The EKF error state formulation is supported by the fact that the predicted state vector at the instant *k* can be expressed as the true state vector plus a certain error. Being **X̃***_k_* the true state vector, **x***_k_* the predicted state vector and *δ***x***_k_* the error state vector:

(1) $${\tilde{\text{X}}}_{k}={\text{x}}_{k}+\delta {\text{x}}_{k}$$

According to Kalman, the predicted state vector at instant (*k* + 1) (e.g., **X̃***_k_*_+1_), assuming a linear system and no control variables, can be expressed as:

(2) $${\tilde{\text{X}}}_{k+1}=A{\tilde{\text{X}}}_{k}+\text{n}$$

where *A* is the state transition model and **n** represents the process noise. If Equation (2) is expressed in terms of Equation (1), then:

(3) $${\text{x}}_{k+1}+\delta {\text{x}}_{k+1}=A{\text{x}}_{k}+A\delta {\text{x}}_{k}+\text{n}$$

Assuming that the vector state has no error or noise, the state transition can be expressed as:

(4) $${\text{x}}_{k+1}=A{\text{x}}_{k}$$

and the error transition as:

(5) $$\delta {\text{x}}_{k+1}=A\delta {\text{x}}_{k}+\text{n}$$

Similarly to [23], the Kalman system will update the error vector, which will be added to the state vector, giving the appearance that the update is applied directly to it.

#### 4.2.2. State Vector and Error State Vector

Two vectors are involved in the process, the nominal state and the error state. Let **x***_k_* be the nominal state vector at time *k*:

(6) $${\text{x}}_{k}=\left({\text{p}}_{k},{\text{q}}_{k},{\text{v}}_{k},\omega_{k},{\text{a}}_{k},{\text{d}}_{k},{\text{b}}_{k}\right)$$

where **p***_k_* is the position of the vehicle with respect to the inertial global frame, **q***_k_* represents its attitude as a quaternion, **v***_k_* is the robot linear local velocity (unlike [21], where the velocity is with reference to the global inertial frame), ***ω****_k_* is the angular rate, **a***_k_* is the linear local acceleration and **d***_k_* and **b***_k_* denote the drift of the gyroscope and the bias of the accelerometer, respectively. The biases present in the acceleration and in the angular rate given by the inertial sensors are assumed to be constant. It is assumed that the nominal state does not include noise or model imperfections.

(7) $${\text{p}}_{k}=(x(k),y(k),z(k))$$

(8) $${\text{q}}_{k}=(q_{x}(k),q_{y}(k),q_{z}(k),q_{w}(k))$$

(9) $${\text{v}}_{k}=(v_{x}(k),v_{y}(k),v_{z}(k))$$

(10) $$\omega_{k}=(\omega_{x}(k),\omega_{y}(k),\omega_{z}(k))$$

(11) $${\text{a}}_{k}=(a_{x}(k),a_{y}(k),a_{z}(k))$$

(12) $${\text{d}}_{k}=(d_{x}(k),d_{y}(k),d_{z}(k))$$

(13) $${\text{b}}_{k}=(b_{x}(k),b_{y}(k),b_{z}(k))$$

Let *δ***x***_k_* be the error state vector at time *k*, including the errors caused by sensor biases and random noise:

(14) $$\delta {\text{x}}_{k}=(\delta {\text{p}}_{k},\delta {\text{q}}_{k},\delta {\text{v}}_{k},\delta \omega_{k},\delta {\text{a}}_{k})$$

where *δ***p***_k_* represent the error in position, *δ***q***_k_* is the error in attitude in the form of a rotation vector, *δ***v***_k_* is the error in the linear local velocity and *δ****ω****_k_* with *δ***a***_k_* are the errors in the angular rate and in the linear local acceleration, respectively, which are also assumed to be constant. Errors in acceleration and in angular rate include sensor biases and noise.

(15) $$\delta {\text{p}}_{k}=(\delta x(k),\delta y(k),\delta z(k))$$

(16) $$\delta {\text{q}}_{k}=(\delta q_{x}(k),\delta q_{y}(k),\delta q_{z}(k))$$

(17) $$\delta {\text{v}}_{k}=(\delta v_{x}(k),\delta v_{y}(k),\delta v_{z}(k))$$

(18) $$\delta \omega_{k}=(\delta \omega_{x}(k),\delta \omega_{y}(k),\delta \omega_{z}(k))$$

(19) $$\delta {\text{a}}_{k}=(\delta a_{x}(k),\delta a_{y}(k),\delta a_{z}(k))$$

An EKF framework usually consists of two steps, a prediction and an update [37]. However, this navigation filter proceeds in four steps:

1. Nominal state prediction: the IMU data are integrated to estimate the vehicle motion and the predicted nominal state.
2. Error state prediction: the error state is computed from the predicted nominal state and from the last error state. The error state values are zero due to the error reset performed in the fourth step, but the covariance matrix is different from zero.
3. Error Kalman update: corrects the predicted error state and covariances with the errors of the different sensor measurements and returns an updated error state vector (now different from zero) with its covariance matrix.
4. Nominal state correction and error reset: the error state vector is used to adjust the nominal state data; afterwards, the error vector is reset to zero.

#### 4.2.3. Nominal State Prediction

The readings of the inertial sensors are used at this stage to estimate the relative vehicle displacement (translation and rotation) between two filter iterations. The inertial data contain a certain bias and noise, assumed to be additive and zero-mean Gaussian.

Let ***ω*** be the true vehicle angular rate given by [23]. At time *k*:

(20) $$\omega_{m}=\omega +\text{d}+{\text{n}}_{\omega }$$

where ***ω****_m_*, **d** and **n***_ω_* denote, respectively, the sensor readings, the drift and the noise of the gyroscope.

The readings of the inertial sensors are usually referenced to the body frame, and the acceleration can include the locally-referenced gravity in the vertical axis. Let us assume, without loss of generality, that the inertial sensor is approximately located in the center of the vehicle, the centripetal acceleration being negligible. At time *k*:

(21) $${\text{a}}_{m}=(\text{a}+{\text{g}}_{L})+\text{b}+{\text{n}}_{a}$$

(22) $${\text{g}}_{L}={\text{R}}^{⊤}\cdot \text{g}$$

where **a***_m_* is the sensor reading, **b** is the accelerometer bias, **n***_a_* is its noise and **a** is the real body acceleration at time *t*. The gravity, **g***_L_*, is expressed with respect to the body frame, and 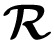 is the rotation matrix from local to global coordinates (corresponding to the quaternion **q**, the vehicle attitude); so 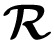
^T^ is the transposed rotation matrix of 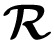, permitting the transform from global to local coordinates, and **g** = (0, 0, −*g*), being *g* = 9.81 m/s^2^.

**Figure 4.**
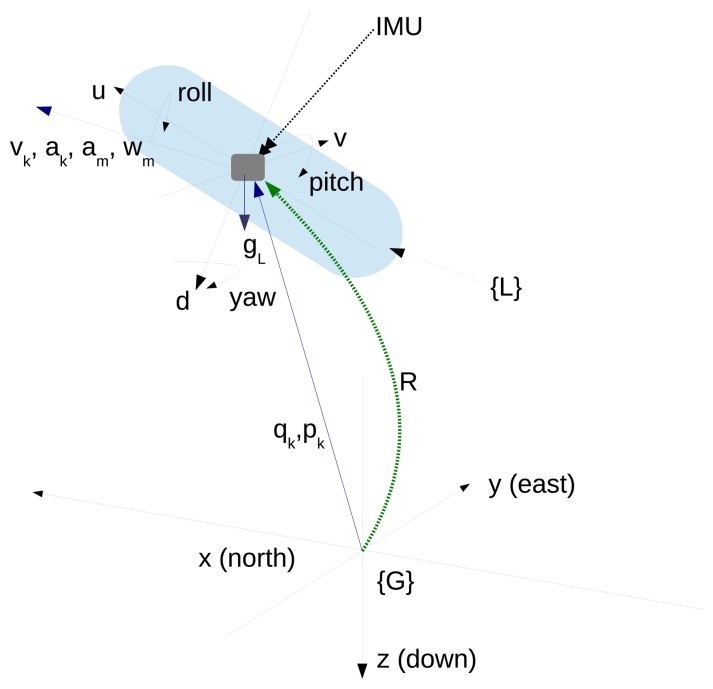
Coordinate frames and notations. {G} is the inertial global frame; {L} is the coordinate frame attached to the vehicle. u,v and d represent the position coordinates referenced to {L}.

Note that **v***_k_*, ***ω****_k_*, **a***_k_*, **d***_k_* and **b***_k_* are referenced to the local coordinate system (e.g., the system of coordinates attached to the vehicle), and **p***_k_* and **q***_K_* are referenced to the inertial frame (e.g., global frame). The coordinate system conventions and notations are illustrated in Figure 4.

In this particular implementation, the discrete equations that govern the nominal state are based on a general constant acceleration motion. Note that *x*_(_*_k_*_−1)_ ≡ *x*_(_*_k_*_−1)|(_*_k_*_−1)_ and that the estimated state at instant *k* dependent on the estimated state at instant *k* − 1 is denoted as *x*_(_*_k_*_)|(_*_k_*_−1)_.

(23) $${\text{p}}_{k|\left(k-1\right)}={\text{p}}_{\left(k-1\right)}+{\text{R}}_{\left(k-1\right)}{\text{v}}_{\left(k-1\right)}\Delta t+\frac{1}{2}{\text{R}}_{\left(k-1\right)}{\text{a}}_{\left(k-1\right)}\Delta t^{2}$$

(24) $${\text{q}}_{k|\left(k-1\right)}={\left({\text{q}}_{m}*\text{q}\right)}_{\left(k-1\right)}$$

(25) $${\text{v}}_{k|\left(k-1\right)}={\text{v}}_{k-1}+{\text{a}}_{\left(k-1\right)}\Delta t$$

(26) $$\omega_{k|\left(k-1\right)}={\left(\omega_{m}-\text{d}\right)}_{\left(k-1\right)}$$

(27) $${\text{a}}_{k|\left(k-1\right)}={\left({\text{g}}_{L}-{\text{a}}_{m}+\text{b}\right)}_{\left(k-1\right)}$$

(28) $${\text{d}}_{k|\left(k-1\right)}={\text{d}}_{\left(k-1\right)}$$

(29) $${\text{b}}_{k|\left(k-1\right)}={\text{b}}_{\left(k-1\right)}$$

where **Δ***t* is the time interval between the current and the predicted nominal state, * represents the quaternion product and **q***_m_* is the quaternion that represents the vehicle angular motion occurring during **Δ***t*.

(30) $${\text{q}}_{m}=\text{quaternion}(\omega_{m}\Delta t)={\left(\begin{array}{c} \cos(\omega_{x}\Delta t/2)\\ 0\\ 0\\ \sin(\omega_{x}\Delta t/2) \end{array}\right)}^{⊤}*{\left(\begin{array}{c} \cos(\omega_{y}\Delta t/2)\\ 0\\ \sin(\omega_{y}\Delta t/2)\\ 0 \end{array}\right)}^{⊤}*{\left(\begin{array}{c} \cos(\omega_{z}\Delta t/2)\\ \sin(\omega_{z}\Delta t/2)\\ 0\\ 0 \end{array}\right)}^{⊤}$$

It is important to observe that, since the acceleration **a***_k_* and the linear velocity **v***_k_* are referenced to the local body frame, the term **g***_L_* present in the expression of **a***_k_* transforms the gravity expressed with respect to the inertial frame into the body frame. However, the accumulated position **p***_k_* must be given with respect to the inertial global frame. As a consequence, the terms 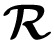
**_(_***_k−_*_1)_**v**_(_*_k_*_−1)_ and 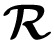
**_(_***_k_*_−1)_**a_(_***_k_*_−1)_ present in the expression of **p***_k_* denote the transformation of the velocity and the acceleration from the body frame to the inertial global frame.

#### 4.2.4. Error State Prediction

The error is defined as the difference between the estimate of a certain variable and its real value. Let *δ***x̃***_k_* be the estimation of the error state vector at time *k*:

(31) $$\delta {\tilde{\text{x}}}_{k|\left(k-1\right)}=f({\text{x}}_{\left(k-1\right)})+\epsilon_{k}$$

where ***f***(**x***_k_*) is the general set of functions defined to predict the error state and ***ϵ****_k_* = *N*(0, *Q_k_*) is the zero-mean Gaussian error of the prediction model, *Q_k_* being the model noise covariance.

Let *δ***ã***_k_*, *δ****ω̃****_k_*, *δ***p̃***_k_*, *δ***q̃***_k_*, δ**ṽ***_k_*, be the estimated errors in acceleration, angular velocity, position, attitude and linear velocity, all at time *k*. The discrete equations ***f***(**x***_k_*_|(_*_k_*_−1_**_)_**) that govern the error state estimation are:

(32) $$\delta {\tilde{\text{p}}}_{k|\left(k-1\right)}=\delta {\tilde{\text{p}}}_{\left(k-1\right)}+\text{R}\delta {\tilde{\text{v}}}_{\left(k-1\right)}\Delta t-\text{R}({\tilde{\text{v}}}_{\left(k-1\right)}⊗\delta {\tilde{\text{q}}}_{\left(k-1\right)})\Delta t-\frac{1}{2}\text{R}({\tilde{\text{a}}}_{\left(k-1\right)}⊗\delta {\tilde{\text{q}}}_{\left(k-1\right)})\Delta t^{2}+\frac{1}{2}\text{R}\delta {\tilde{\text{a}}}_{\left(k-1\right)}\Delta t^{2}$$

(33) $$\delta {\tilde{\text{v}}}_{k|\left(k-1\right)}=\delta {\tilde{\text{v}}}_{\left(k-1\right)}+{\text{g}}_{L}⊗\delta {\tilde{\text{q}}}_{\left(k-1\right)}\Delta t+\delta {\tilde{\text{a}}}_{\left(k-1\right)}\Delta t$$

(34) $$\delta {\tilde{\omega }}_{k|\left(k-1\right)}=\delta {\tilde{\omega }}_{k-1}$$

(35) $$\delta {\tilde{\text{a}}}_{k|\left(k-1\right)}=\delta {\tilde{\text{a}}}_{k-1}$$

(36) $$\delta {\tilde{\text{q}}}_{k|\left(k-1\right)}={\text{R}}_{m}\delta {\tilde{\text{q}}}_{\left(k-1\right)}+\left(-{\text{I}}_{3\times 3}\Delta t+\frac{1-\cos(\left|\omega_{\left(k-1\right)}\right|\Delta t)}{{\left|\omega_{\left(k-1\right)}\right|}^{2}}\text{V}-\frac{\left|\omega_{\left(k-1\right)}\right|\Delta t-\sin(\left|\omega_{\left(k-1\right)}\right|\Delta t)}{{\left|\omega_{\left(k-1\right)}\right|}^{3}}{\text{V}}^{2}\right)\delta {\tilde{\omega }}_{\left(k-1\right)}$$

where 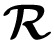
*_m_* is the rotation matrix corresponding to ***ω**_m_*Δ*t*, also depending on time (*k −* 1). Note that *δ***ã***_k_*_|(_*_k_*_−1)_ and *δ****ω****˜*_*k*|(*k*−1)_ are supposed as constant, so that the filter can find the constant bias and drift of the sensors. **V** is the skew symmetric matrix of the velocity, in the form:

(37) $$\text{V}=\left(\begin{array}{ccc} 0 & -v_{z} & v_{y}\\ v_{z} & 0 & -v_{x}\\ -v_{y} & v_{x} & 0 \end{array}\right)$$

**I**_3×3_ is the three by three identity matrix and ⊗ represents the cross product.

Derivation of Equation (36) can be found in [35], and derivations of Equations (32) and (33) can be found in the Appendix of this paper.

As is exposed in Section 4.2.6, the values forming the error state are all reset after the nominal state has been corrected, and thereby, the values computed by Equations (32) to (36) are always zero. However, the corresponding Jacobian ${F_{k}=\frac{\partial f(x_{k})}{\partial \delta (x_{k})}|}_{\delta {\tilde{x}}_{k}}$, at iteration *k* is different from zero. Therefore, the system covariance matrix **P***_k_* can be propagated according to the Kalman equation:

(38) $${\text{P}}_{k|\left(k-1\right)}={\text{F}}_{k}{\text{P}}_{(k-1)|\left(k-1\right)}{\text{F}}_{k}^{⊤}+{\text{Q}}_{k}$$

#### 4.2.5. Error Kalman Update

Let us denote the measurement error *δ***z***_k_* as the difference between a sensor reading **z***_k_* and its prediction **z̃***_k_* contained in the nominal state:

(39) $$\delta {\text{z}}_{k}={\text{z}}_{k}-{\tilde{\text{z}}}_{k}$$

According to a standard EKF, the observation function *h*(*δ***x̃***_k_*) will be used to compute the predicted error measurement from the predicted error state. In this particular design of ESKF, and similarly to [21], the observation function is assumed to be a linear approximation and stated as *h*(*δ***x̃***_k_*) = **H***_k_δ***x̃**_*k*|__(_*_k_*_−1)_ , **H***_k_* being the identity matrix. This is the same as assuming that, by definition, the measurement error is predicted as exactly the value of the estimated error state. Then, the innovation can be written as:

(40) $${\tilde{\text{y}}}_{k}=\delta {\text{z}}_{k}-{\text{H}}_{k}\delta {\tilde{\text{x}}}_{k|\left(k-1\right)}$$

being *δ***z***_k_* the vector of measurement errors.

In this step, the classical Kalman update equations are used to obtain an updated error state vector (δ**x̃***_k_*) and its covariance, now both different from zero:

(41) $${\text{S}}_{k}={\text{H}}_{k}{\text{P}}_{k|\left(k-1\right)}{\text{H}}_{k}^{⊤}+{\text{R}}_{k}$$

(42) $${\text{K}}_{k}={\text{P}}_{k|\left(k-1\right)}{\text{H}}_{k}^{⊤}{\text{S}}_{k}^{-1}$$

(43) $$\delta {\tilde{\text{x}}}_{k|k}=\delta {\tilde{\text{x}}}_{k|\left(k-1\right)}+{\text{K}}_{k}{\tilde{\text{y}}}_{k}$$

(44) $${\text{p}}_{k|k}=(\text{I}-{\text{K}}_{k}{\text{H}}_{k}){\text{P}}_{k|\left(k-1\right)}$$

**S***_k_* being the innovation covariance, **K***_k_* the Kalman gain and **R***_k_* the measurements uncertainty. Note the difference between this **R***_k_* and the rotation matrix 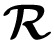 defined in Section 4.2.3.

#### 4.2.6. Nominal State Correction and Error Reset

The values obtained in the updated error state vector are used to correct the nominal state vector by Equations (45)–(51).

(45) $${\text{p}}_{k|k}={\text{p}}_{k|\left(k-1\right)}+\delta {\tilde{\text{p}}}_{k|k}$$

(46) $${\text{q}}_{k|k}={\text{q}}_{k|\left(k-1\right)}*\text{quaternion}(\delta {\tilde{q}}_{k|k})$$

(47) $${\text{v}}_{k|k}={\text{v}}_{k|\left(k-1\right)}+\delta {\tilde{\text{v}}}_{k|k}$$

(48) $$\omega_{k|k}=\omega_{k|\left(k-1\right)}-\delta {\tilde{\omega }}_{k|k}$$

(49) $${\text{a}}_{k|k}={\text{a}}_{k|\left(k-1\right)}+\delta {\tilde{\text{a}}}_{k|k}$$

(50) $${\text{d}}_{k|k}={\text{d}}_{k|\left(k-1\right)}+\delta {\tilde{\omega }}_{k|k}$$

(51) $${\text{b}}_{k|k}={\text{b}}_{k|\left(k-1\right)}+\delta {\tilde{\text{a}}}_{k|k}$$

It is important to notice that *quaternion*(*δ***q̃***_k|k_*) represents the quaternion corresponding to the rotation vector *δ***q̃***_k|k_*. Contrary to the rest of nominal variables, the angular velocity is decremented with its corresponding error. Equation (48) is derived from Equation (20), where the true vehicle angular rate is expressed in terms of the measurement, drift and noise:

(52) $$\omega =\omega_{m}-\text{d}-{\text{n}}_{\omega }$$

now with time indexes, and underestimating the error term,

(53) $$\omega_{k|k}=\omega_{m_{k|k}}-{\text{d}}_{k|k}$$

substituting **d***_k_*_|_*_k_* with the correction Equation (50),

(54) $$\omega_{k|k}=\omega_{m_{k|k}}-{\text{d}}_{k|k-1}-\delta {\tilde{\omega }}_{k|k}$$

but as there is only one measure per iteration, ***ω***_*m*_*k*|*k*__ = ***ω***_*m*_*k*|(*k*−1)__, then substituting with the prediction term in Equation (26),

(55) $$\omega_{k|k}=\omega_{k|k-1}-\delta {\tilde{\omega }}_{k|k}$$

This filter corrects the whole nominal state, including the biases of the inertial sensor, contrary to other solutions based on indirect Kalman filters that do not include sensor biases in the state vector [38], or where the acceleration is estimated externally of the filter state [19].

As indicated in [23], after this correction, all variables of the error state vector are reset to zero, and the algorithm starts again the next iteration in Step 1 with the corrected value of **x***_k_*. This reset does not modify the covariance, since it does not alter the content of the Kalman filter estimates. It merely moves the data from one vector to the other, avoiding the need to propagate two state vectors and limiting the covariance variations to the error dynamics.

#### 4.2.7. Measurement Delays

The different sensors that equip Fugu-C supply their data at different rates. While the operational frequency of an standard IMU can range from 100 Hz to 1 KHz, a visual odometer or a pressure sensor can work, typically, between 10 Hz and 25 Hz (depending on the capacity of the used processor).

Motion predictions are done at the IMU transmission rate, while updates must be run when one exteroceptive measurement is received. The lack of synchronism in the integration of the data coming from the diverse sensors, that is delays between the reception and the processing of the IMU or odometry messages, and delays in the execution of the prediction and update steps can introduce additional errors in the filtering process.

To assure that all of the measurements are incorporated synchronously to the filter and that every update is launched synchronously with its corresponding motion prediction, all of the sensor timestamps are referred to the same global timer. Furthermore, similarly to [22], all of the measurements are stored in FIFO buffers with their timestamp. Although every measurement can really be processed with a certain delay, the updates are executed, aligned in time with the corresponding prediction, which is the one performed with the IMU reading that has a time stamp that immediately precedes the time stamp of the currently-processed visual sample.

It is worth commenting that, although the dynamics of Fugu-C are quite slow and this problem would not really affect the vehicle control, the filter has a general design, and it is prepared also to work in vehicles with six DOF with faster dynamics. This issue is crucial, for instance, in UAVs.

**Figure 5.**
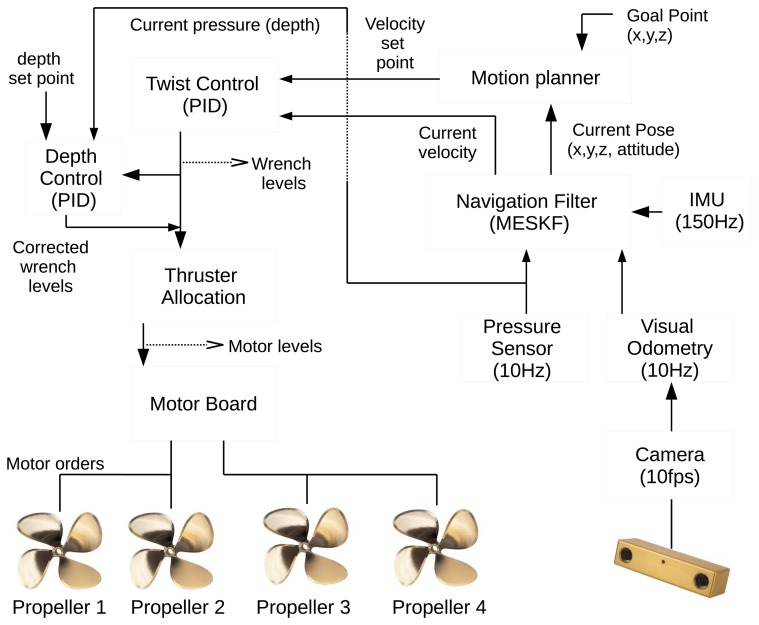
The navigation and control architecture.

Our implementation of the MESKF and its ROS wrapper is available to the scientific community in a public repository [26]. Figure 5 shows a schematic overview of the ROS node-based navigation architecture described in this section.

## 5. Experimental Results

The experimental results are organized in experiments in a controlled environment, experiments in the sea and 3D reconstruction.

### 5.1. Navigation in a Controlled Environment

**Figure 6.**
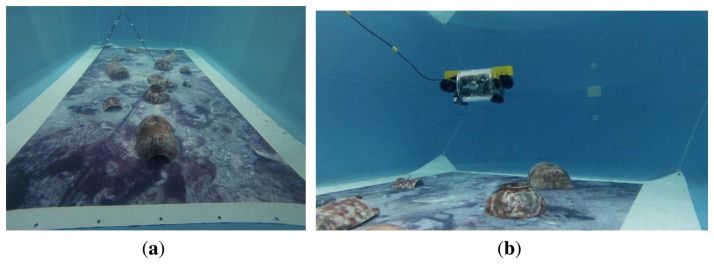
(**a**) The poster covering the pool bottom, with some artificial reliefs on it; (**b**) Fugu-C navigating in the pool.

The first set of experiments to assess the navigation modules was carried out actuating the robot at a constant depth in a pool of 7 × 4 m. The bottom of this pool was covered with a printed digital image of a sea bottom environment, to simulate a marine-like context. In order to have a certain reference, the poster was also used to calculate the trajectory ground truth (see Figure 6). To that end, features of the online stereo images, captured from the down-looking camera, are matched with those features extracted offline from the original digital image of the poster. Afterwards, the 3D points computed from the online stereo pairs are re-projected onto the matching features of the original digital image, trying to minimize the global re-projection error. This is a particular application of the perspective-N-point (PNP) problem [39], and the result of the algorithm is the relative transformation between the current stereo image pair and the corresponding section in the original image of the poster. The algorithm assumes a flat ground and a perfect alignment of 90° between the lens axis of both cameras and the ground surface. The precision in the computation of the ground truth under the ideal conditions is high, since the outliers are eliminated using random sample consensus (RANSAC) [40], and errors in the re-projection and optimization process are minimized.

**Figure 7.**
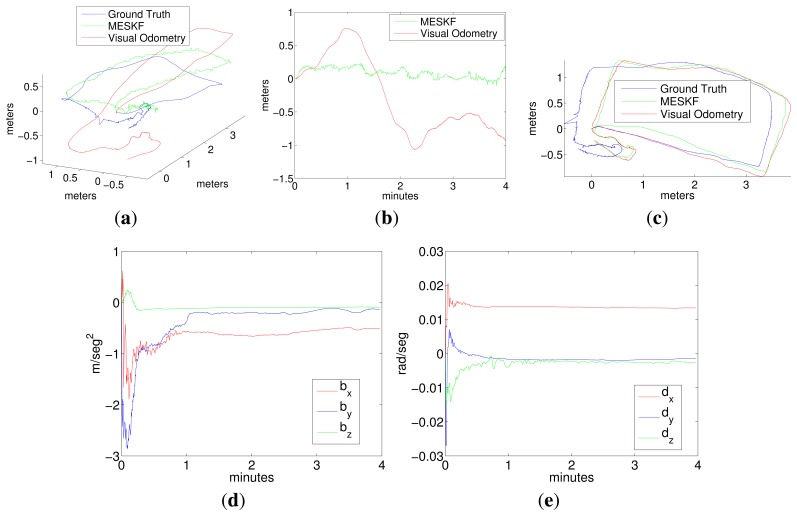
Experiments 1. (**a**) Robot trajectory in 3D; (**b**) depth; (**c**) robot trajectory in the x-y plane; (**d**) acceleration bias; (**e**) gyro drift.

**Figure 8.**
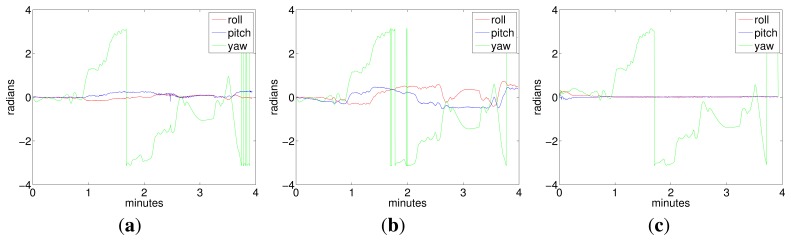
Vehicle attitude corresponding to Experiment 1. (**a**) Ground truth; (**b**) visual odometry; (**c**) multiplicative error state Kalman filter (MESKF).

Two representative experiments in the pool are shown in Figures 7, 8 (a loop trajectory) and 9 (a sweeping trajectory).

The origin of the global coordinate system is always set at the beginning of the trajectory. This point is located at the water surface (*z* = 0), so the depth computed from the pressure sensor is equivalent to the *z* > 0 axis. The initial 15 × 15 covariance matrix of the error state vector was set to zero in all of the positions, except in the diagonal, where all elements were set to small values (10^−2^) to represent small uncertainties in the initial state vector.

Plot 7a shows the trajectory in (x,y,z) for the visual odometry (red), ground truth (blue) and for the filter estimates (green), and Plot 7c shows the same robot trajectory in the x-y plane.

Plot 7d shows the evolution of the accelerometer bias returned by the filter, and Plot 7e shows the evolution of the gyro drift.

Figure 8 shows the vehicle attitude during Experiment 1, corresponding to the ground truth (Plot 8a), the visual odometry (Plot 8b) and to the filter (Plot 8c).

**Figure 9.**
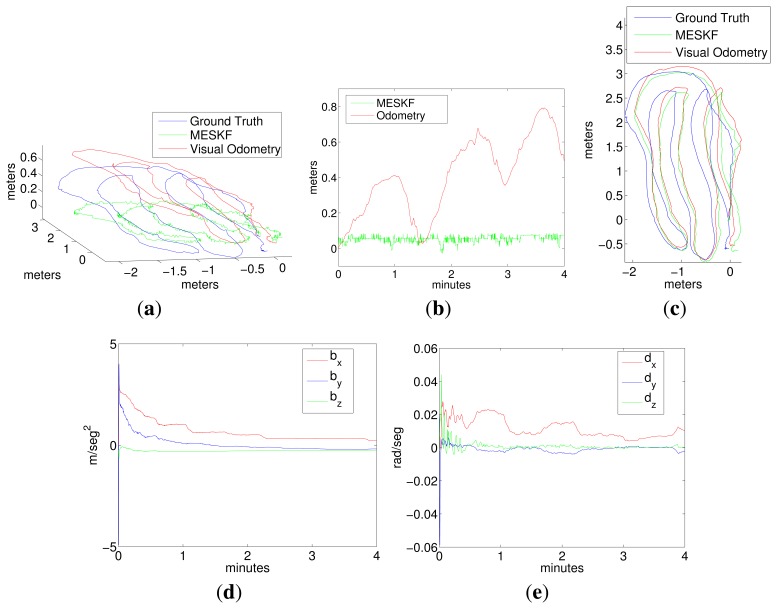
Experiment 2. A sweeping trajectory. (**a**) Robot trajectory in 3D; (**b**) depth; (**c**) robot trajectory in the x-y plane; (**d**) acceleration bias; (**e**) gyro drift.

**Figure 10.**
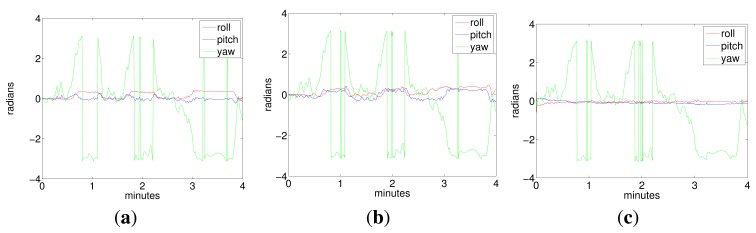
Vehicle attitude corresponding to Experiment 2. (**a**) ground truth; (**b**) visual odometry; (**c**) MESKF.

Figure 9a,c shows, respectively, the 3D and 2D views of a sweeping trajectory run in the second experiment. Figure 9b shows the depth of the vehicle according to the visual odometry and the filter. Again, the origin of the trajectory is in the point (0, 0, 0). Plots 9d and 9e show the accelerometer bias and the gyro drift, respectively. Figure 10 shows the vehicle orientation in roll, pitch and yaw, according to the ground truth (Plot a), the visual odometry (Plot b) and the filter estimates (Plot c).

Results of both experiments point out the next conclusions:

- It can be observed from trajectories shown in Figures 7a and 9a that, although the data from the visual odometer present an important drift in *z*, the filter is able to correct this coordinate to a nearly constant value, using the pressure sensor information. The trajectory estimated by the filter is very close to the visual odometer, which is, in fact, much more reliable than the position estimated by integrating the inertial data. It is important to remark that the goal of the filter is not the pose correction. Since this is an approach for inertially-aided navigation, here, there are no external landmarks or loop closings used to improve the robot position further than the odometer estimates. Instead, the aim is to integrate all of the data coming from the navigation sensors in the most convenient way to, on the one hand, continuously obtain a vehicle pose that is as reliable as possible and, on the other hand, to stabilize the inertial data, compensating for their systematic biases.
- Acceleration bias and gyro drift stabilize quickly to constant values (some of them close to zero), meaning a practical sensor calibration and permitting the compensation of the inertial systematic errors. The highest value in the gyro drift corresponds to approximately 0.015 rad in its *x* component, an imperceptible value compared with the typical drifts that standard inertial sensors and odometers are prone to reach. Since the vehicle is moving at, approximately, a constant depth, (oscillating slightly in *z* due to the PID vertical hysteresis), the acceleration in *z* is very small and so is its drift in *z*.
- All of the trajectories estimated by the filter present, at their origin, a difference in orientation with respect to the ground truth. This difference is caused by the initial error in the angular rate. However, as this initial error is rapidly compensated for by the filter, it is not accumulated in the subsequent pose estimates.
- As Fugu-C is passively stable in pitch and roll, motion in these two DOF should result in being insignificant, while rotations in yaw should reflect the heading changes at every turn. In all of the trajectories, the vehicle always moved only in surge, heave and yaw. Consequently, the values corresponding to pitch and roll should be very small. However, the values of pitch and roll estimated by the ground truth and by the visual odometer in Experiments 1 and 2, as shown in Figures 8a,b and 10a,b, present values significantly different from zero (with maxima in the odometry of 0.5 radians), which is incorrect. The ground truth also presents, erroneously, non-negligible values of roll and pitch. This is because the condition of flat ground is not completely fulfilled due to a slight inclination of the pool bottom with respect to the water surface. Furthermore, the alignment of the lens axis of the down-looking camera with the normal to the ground plane is not perfect. Conversely, the filter approaches the values of roll and pitch to zero during both trajectories, which is much closer to the vehicle's real behavior. The ground truth in these experiments performs well in (*x*, *y*), and it is a reliable reference of the vehicle trajectory in the horizontal plane. However, unfortunately, it presents some errors in the orientation estimates and in the vertical direction.

### 5.2. Navigation in the Sea

Other experiments were conducted in a shallow water area off the coast of Mallorca (Balearic Islands, Spain). The robot was actuated only in surge, heave and yaw, without any significant motion in roll or pitch. Fugu-C was programmed to navigate at a constant depth in four different trajectories: one L-shaped, running close to a harbor breakwater, a loop and two sweeping trajectories covering around 40 meters of length. The sea bottom in the experiment area had no significant slope and was mainly covered by rocks and algae. Local reliefs caused significant and frequent changes in the distance from the vehicle to the ground. Even so, the experiments demonstrate that the filter responds properly, compensating for the visual odometer drift with the pressure sensor data. For these experiments, there was no possibility to calculate a ground truth. Instead, several markers were put on the sea bottom to control the difference between the real markers' position and the estimated pose of the vehicle when it passed over them. All trajectories started at the same point, indicated with a marker. Nonetheless, the goal of the filter is not a pose correction, but there is the self-calibration of the inertial sensors.

Besides, taking advantage of the *rosbag* [25] technology offered by the ROS middleware, the results of Experiments 3, 4 and 5, obtained with our MESKF, were compared with the results obtained using a standard approach of multiplicative extended Kalman filter (MEKF). The node *ekf_localization* of the ROS package *robot_localization* [41] was used to that end. This node implements an multiplicative (orientations represented with quaternions) EKF that permits the integration of six-DOF nominal data coming from several sensors (in our case, the acceleration and the angular rate of an IMU, the pose and twist of a visual odometer and the depth given by the pressure sensor) and returns a refined state vector composed of pose and twist. In the standard EKF, the nominal variables, all affected by additive errors, are inserted in the Kalman filter, while in the MESKF, only the error state is filtered, but its result is added to the nominal state. Both systems should lead to similar results, because both include, integrate, estimate and evolve the error of the system model and the nominal data in different ways. However, the main differences between both approaches are: (1) no error variables are included in the EKF state vector; consequently, the systematic errors of the inertial sensors are not compensated for; (2) there are no significant differences in the estimated 3D robot trajectory; but, since the drift of the gyroscope is not corrected, appreciable irregularities in the returned orientations are obtained with the standard EKF.

**Figure 11.**
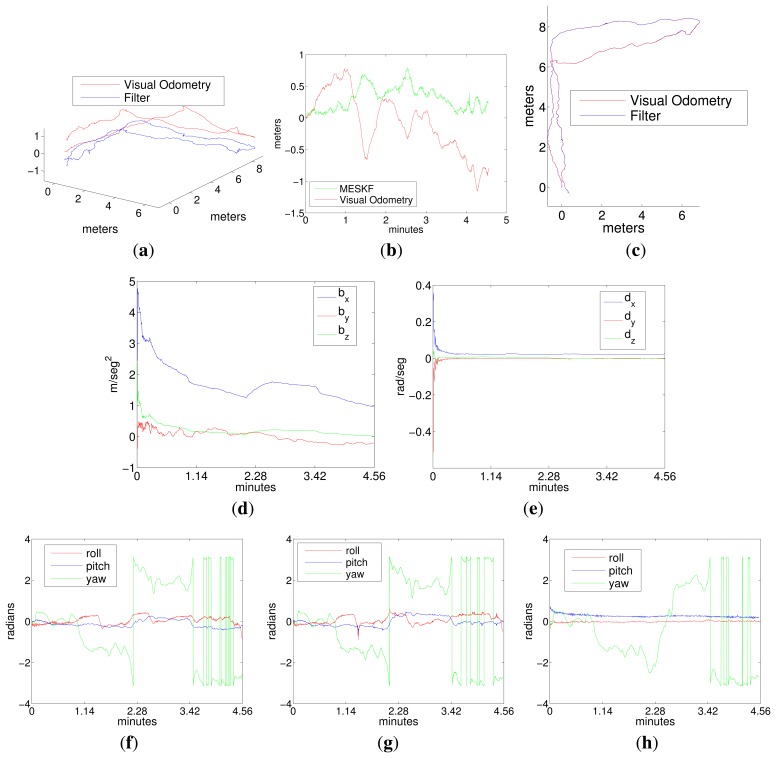
Experiment 3 in the sea using LibViso2 as the stereo odometer. An L-shaped trajectory. (**a**) Robot trajectory in 3D; (**b**) depth; (**c**) robot trajectory in the x-y plane; (**d**) acceleration bias; (**e**) gyro drift; (**f**) vehicle attitude according to the odometer; (**g**) vehicle attitude according to the robot operating system (ROS) EKF [41]; (**h**) vehicle attitude according to our MESKF.

Figure 11 corresponds to an L-shaped trajectory (Experiment 3). The figure shows the trajectory in 3D and 2D, the depth, the acceleration bias, gyro drift and the robot orientation according to the visual odometer and the filter. This figure also shows the vehicle orientation returned by the ROS EKF package [41].

**Figure 12.**
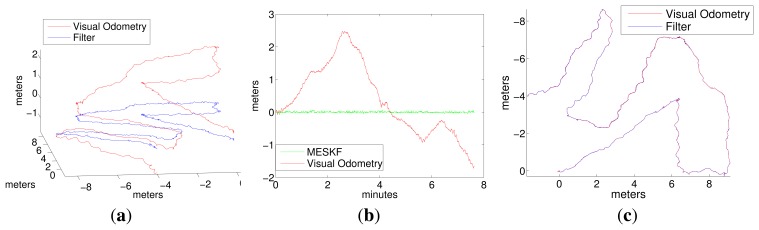

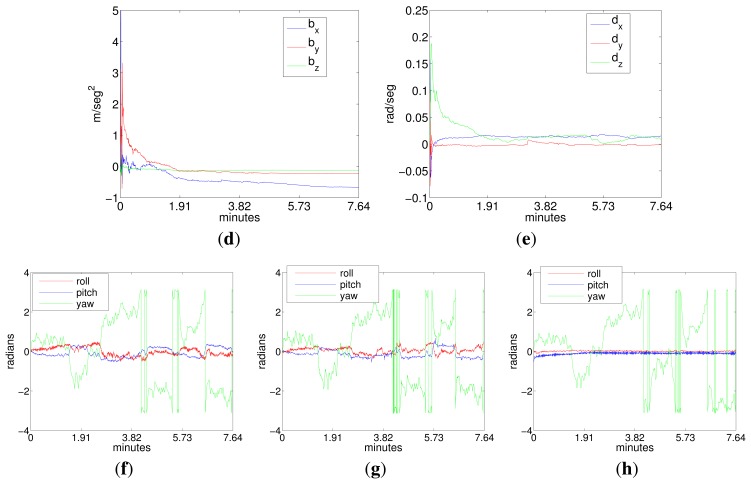
Experiment 4 in the sea using LibViso2 as the stereo odometer. A sweeping trajectory. (**a**) Robot trajectory in 3D; (**b**) depth; (**c**) robot trajectory in the x-y plane; (**d**) acceleration bias; (**e**) gyro drift; (**f**) vehicle attitude according to the odometer; (**g**) vehicle attitude according to the ROS EKF [41]; (**h**) vehicle attitude according to our MESKF.

**Figure 13.**
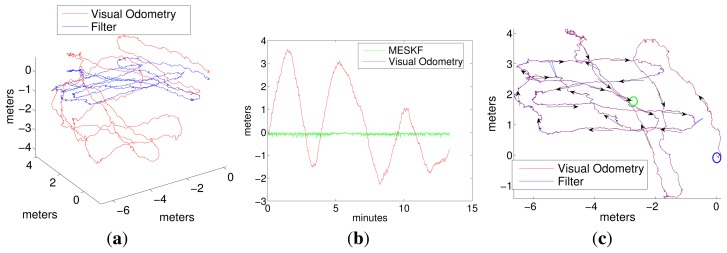

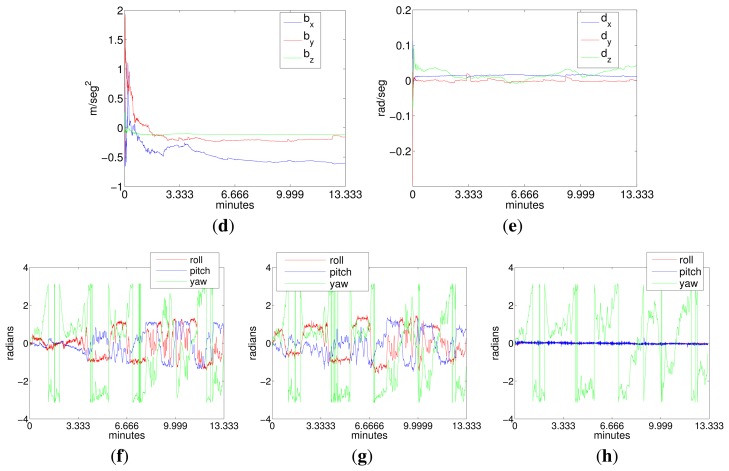
Experiment 5 in the sea using LibViso2 as the stereo odometer. A longer sweeping trajectory. (**a**) Robot trajectory in 3D; (**b**) depth; (**c**) robot trajectory in the x-y plane; (**d**) acceleration bias; (**e**) gyro drift; (**f**) vehicle attitude according to the odometer; (**g**) vehicle attitude according to the ROS EKF [41]; (**h**) vehicle attitude according to our MESKF.

**Figure 14.**
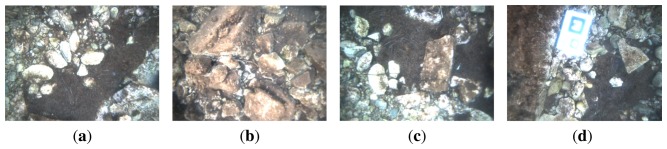
Figures (**a**–**d**) show some images gathered during the experiments in the sea. (d) also shows the artificial marker that indicates the stating point.

Figures 12 and 13 correspond to the two sweeping trajectories conducted in the sea (Experiments 4 and 5). Both figures show the trajectory in 3D and 2D, the depth and the robot orientation, both according to LibViso2 and the MESKF, as well as the acceleration bias and the gyro drift given by the filter. These figures also show the vehicle orientation returned by the ROS EKF package [41]. The length of both experiments exceeds 40 meters. Figure 14 shows four images captured during Experiments 3, 4 and 5. Figure 14d shows the artificial marker deposited at the sea bottom to indicate the starting/end point of the trajectory.

The results of these experiments lead to the next discussion:

- The drift in *z* generated by the visual stereo odometer was corrected by including the pressure sensor data in the filter. In these experiments, the difference between the odometry and the filter estimates in the (x,y) plane is nearly zero. Some settings in the sea are slightly different than in the pool, for example the initial error state covariances and, especially, the odometry covariances (given directly by the visual odometer library and clearly influenced by the type of environment and, thus, the features found in it) and the covariances of the inertial data, which differ from those obtained in the pool, since the motion of the vehicle is conditioned by the slight sea current and the actions of the vehicle PID to compensate for this.
- Trajectories given by the ROS EKF have not been printed, because they are nearly equal to the trajectories estimated by the visual odometer; similar to the odometer, the vehicle attitude estimated by the ROS EKF has non-negligible values, with some maxima and minima close to 0.5 rad, which are not adjusted to its real behavior.
- The orientation of the vehicle in roll and pitch was clearly adjusted by the MESKF to values close to zero, thanks to the rapid compensation of the gyro drift.
- The acceleration biases given by the MESKF approximate a constant; the desirable situation would be to obtain biases stabilized to zero for motions at constant speed; however, in the sea, accelerations in the *x* axis, which is the direction of progress, are prone to appear periodically, since the vehicle is constantly trying to overcome the effects of the currents by applying, from time to time, in the forward direction, a certain force given by the PID controller; this problem is not so evident in the pool, where it is much easier to keep the vehicle moving at a constant velocity without interferences.

**Figure 15.**
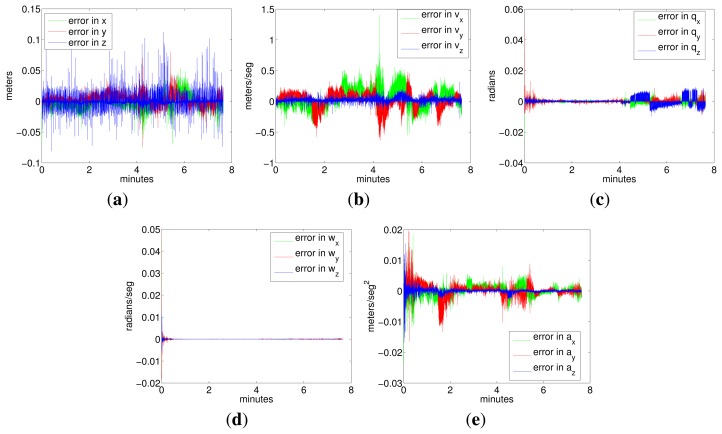
Values of the error state variables during Experiment 4. (**a**) Error in position; (**b**) error in linear velocity; (**c**) error in attitude; (**d**) error in angular rate; (**e**) error in linear acceleration.

Figure 15 shows the evolution of the variables included in the error state vector during Experiment 4. All errors are bounded in a small range: position errors are bounded between ±10 cm; velocity errors are bounded between ±0.5 m/s (except a sample that reaches 1.5 m/s); orientation errors are bounded between approximately ±0.01 radians (0.6°); errors in angular rate are negligible; and errors in acceleration are bounded between approximately 0.02 rad/seg^2^ and −0.015 rad/seg^2^. Values of errors in angular rate and orientation (which include biases and noise) are remarkably small, which means that, according to the previous discussions, attitudes estimated by the filter are highly reliable and much closer to reality than those given by the odometry and by the ground truth algorithm used in this work.

### 5.3. 3D Reconstruction

Stereo video sequences grabbed in the Port of Valldemossa in the ROS-bag [25] format were played offline to simulate the process of building online the 3D map of the environment by concatenating the successive point clouds. The dense point cloud generation was performed at the same rate as the image grabber (10 frames/s), permitting the reconstruction of the environment in real time. The correction in the vehicle estimated attitude increases the precision in the assembly of these point clouds, resulting in a realistic 3D view of the scenario where the robot is moving.

**Figure 16.**
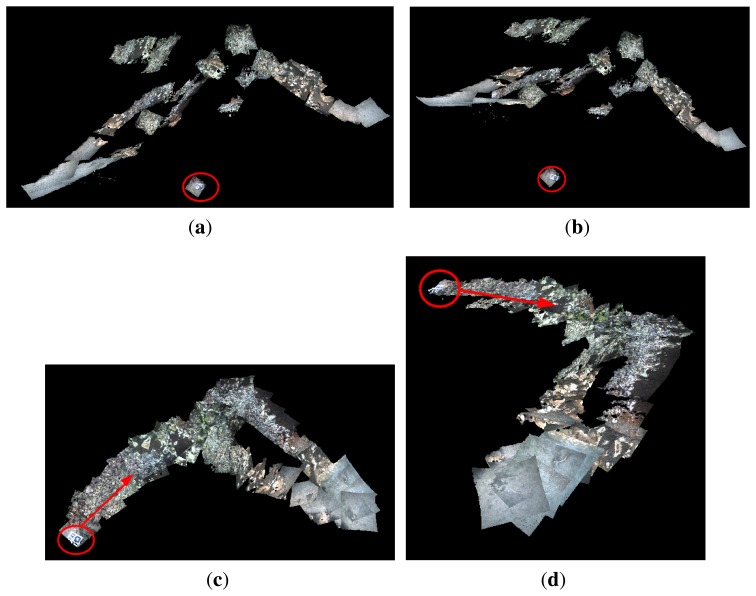
Experiment 3. (**a**,**b**) Two different views of the environment reconstructed in 3D, using the odometry to concatenate the point clouds; (**c**,**d**) two different 3D views of the environment concatenating the point clouds according to the MESKF estimates. In all cases, the red circle indicates the starting point where the marker was deposited, and the red arrow indicates the direction of motion.

Figure 16a,b shows two different 3D views of the marine environment where Experiment 3 was performed. The successive point clouds were registered using the vehicle odometry pose estimates. Figure 16c,d shows two 3D views of the same environments, but registering the point clouds using the vehicle pose estimates provided by the MESKF. In all figures, the starting point and the direction of motion are indicated with a red circle and a red arrow, respectively. A marker was placed in the ground to indicate the starting/end point.

**Figure 17.**
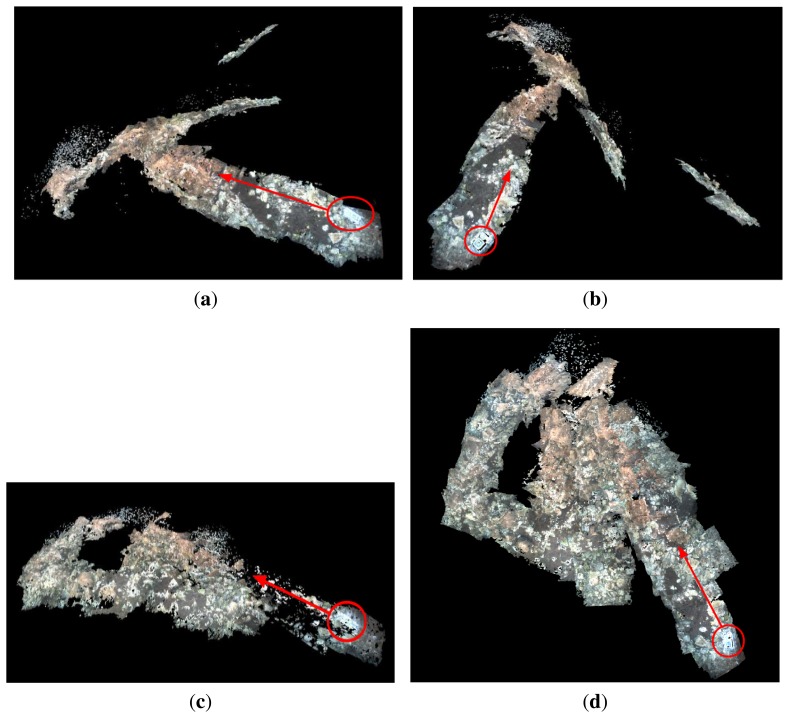
Experiment 4. (**a**,**b**) Two different views of the environment reconstructed in 3D, using the odometry to concatenate the point clouds; (**c**,**d**) two different 3D views of the environment concatenating the point clouds according to the MESKF estimates. In all cases, the red circle indicates the starting point where the marker was deposited, and the red arrow indicates the direction of motion.

Figure 17a,b shows two different 3D views built during Experiment 4, registering the successive point clouds with the vehicle odometry pose estimates. Figure 17c,d shows two 3D views of the same environments, but registering the point clouds using the vehicle pose estimates provided by the MESKF. Again, in all figures, the starting point and the direction of motion are indicated with a red circle and a red arrow, respectively.

**Figure 18.**
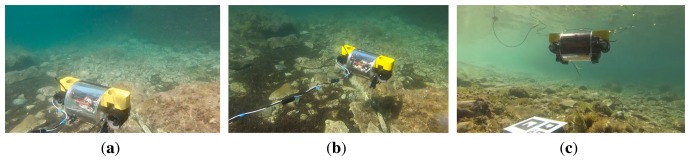
Images (**a**–**c**) show three different views of Fugu-C navigating in the Port of Valldemossa.

Figure 18 shows three images of Fugu-C navigating in this environment. In Figure 18c, the artificial marker deposited on the sea ground can be observed at the bottom of the image.

Raw point clouds are expressed with respect to the camera frame, but then, they are transformed to the global coordinates frame by composing their camera (local) coordinates with the estimated vehicle global coordinates. 3D maps of Figures 16a,b and 17a,b show clear misalignments between diverse point clouds. These misalignments are due to the values of the estimated roll, and pitch vehicle orientations are, at certain instants, significantly different from zero. As a consequence, the orientation of the point cloud has a value different from zero, causing them to be inclined and/or displaced with respect to those immediately contiguous and causing also the subsequent point clouds to be misaligned with respect to the horizontal plane. This effect is particularly evident in Figure 16a,b, where very few point clouds are parallel to the ground, most of them being displaced and oblique with respect to the ground.

However, as the vehicle orientations in roll and pitch estimated by the MESKF are all approximately zero, all of the point clouds are nearly parallel to the ground plane, without any significant inclination in pitch/roll or important misalignment, and providing a highly realistic 3D reconstruction. Notice how the 3D views shown in Figures 16c,d and 17c,d coincide with the trajectories shown in Figures 11 and 12, respectively.

The video uploaded in [42] shows different perspectives of the 3D map built from the dataset of Experiment 4, registering the point clouds with the odometry (at the left) and with the filter estimates (at the right). Observing the 3D reconstructions from different view points offers a better idea of how sloping, misaligned and displaced with respect to the ground, can be some of the point clouds, due to those values of roll and pitch different from zero. The improvement in the 3D map structure when using the filtered data is evident, as all of the point clouds are placed consecutively, totally aligned and parallel to the sea ground.

## 6. Conclusions

This paper presents Fugu-C, a prototype micro-AUV especially designed for underwater image recording, observation and 3D mapping in shallow waters or in cluttered aquatic environments. Emphasis has been made on the vehicle structure, its multiple-layer navigation architecture and its capacity to reconstruct and map underwater environments in 3D. Fugu-C combines some of the advantages of a standard AUV with the characteristics of the micro-AUVs, outperforming other micro underwater vehicles in: (1) its ability to image the environment with two stereo cameras, one looking downwards and another one looking forward; (2) its computational and storage capacity; and (3) the possibility to integrate all of the sensorial data in a specially-designed MESKF that has multiple advantages.

The main benefits of using this aforementioned filter and their particularities with respect to other similar approaches are:

(1) A general configuration permitting the integration of as many sensors as needed and applicable in any vehicle with six DOF.
(2) It deals with two state vectors, the nominal and the error state; it represents all nominal orientations in quaternions to prevent singularities in the attitude estimation; however, the attitude errors are represented as rotation vectors to avoid singularities in the covariance matrices; the prediction model assumes a motion with constant acceleration.
(3) The nominal state contains the biases of the inertial sensors, which permits a practical compensation of those systematic errors.
(4) Linearization errors are limited, since the error variables are very small and their variations much slower than the changes on the nominal state data.
(5) This configuration permits the vehicle to navigate by just integrating the INS data when either the aiding sensor or the filter fail.

Extensive experimental results in controlled scenarios and in the sea have shown that the implemented navigation modules are adequate to maneuver the vehicle without oscillations or instabilities. Experiments have also evidenced that the designed navigation filter is able to compensate, online, the biases introduced by the inertial sensors and to correct errors in the vehicle *z* coordinate, as well as in the roll and pitch orientations estimated by a visual odometer. These corrections in the vehicle orientation are extremely important when concatenating stereo point clouds to form a realistic 3D view of the environment without any misalignment.

Furthermore, the implementation of the MESKF is available to the scientific community in a public repository [26].

Future work plans are oriented toward the following: (1) the aided inertial navigation approach presented in this paper is unable to correct the vehicle position in (*x*, *y*), since it is not using any technique to track environmental landmarks or to adjust the localization by means of closing loops; the use of stereo GraphSLAM [43] to correct the robot position estimated by the filter will be the next step, applying, afterwards, techniques for fine-point cloud registration when they present overlap; (2) obviously, the twist and depth simple PID controllers described in Section 4.1 could be changed by other, more sophisticated systems that take into account other considerations, such as external forces, hydrodynamic models and the relation with the vehicle thrusters and its autonomy; one of the points planned to be investigated in forthcoming work consists of trying to find a trade off between controlling the vehicle only with the navigation sensorial data and the incorporation of a minimal number to structural considerations.

## Appendix

### A. Velocity Error Prediction

Let **ṽ***_k_* be the estimated linear velocity defined according to Equation (25) as:

(A1) $${\tilde{\text{v}}}_{\left(k+1\right)}={\tilde{\text{v}}}_{k}+{\tilde{\text{a}}}_{k}\Delta t$$

where **ã***_k_* is the estimated acceleration.

According to Equations (21) and (A1), it can be expressed as:

(A2) $${\tilde{\text{v}}}_{\left(k+1\right)}={\tilde{\text{v}}}_{k}+({\tilde{\text{R}}}_{k}^{⊤}\text{g}-{\text{a}}_{m}+{\tilde{\text{b}}}_{k})\Delta t$$

and according to Equations (1) and (A2), it can be expressed as:

(A3) $${\text{v}}_{\left(k+1\right)}+\delta {\text{v}}_{\left(k+1\right)}={\text{v}}_{k}+\delta {\text{v}}_{k}+(\delta {\text{R}}_{k}^{⊤}{\text{R}}_{k}^{⊤}\text{g}-{\text{a}}_{m}+{\text{b}}_{k}+\delta {\text{b}}_{k})\Delta t$$

where all of the estimated variables have been substituted by the sum of their nominal and error values, except the rotation matrix, which has to be denoted as a product of the nominal rotation and the rotation error matrices.

The rotation error matrix $\delta {\text{R}}_{k}^{⊤}$ is given by its Rodrigues formula [44], which, for small angles, can be approximated as: $\delta {\text{R}}_{k}^{⊤}\approx I_{3\times 3}+{\left[K\right]}_{x}$, where [*K*]*_x_* is a skew symmetric matrix that contains the rotation vector corresponding to $\delta {\text{R}}_{k}^{⊤}$ and *I*_3×3_ is the 3 × 3 identity matrix.

Consequently, Equation (A3) can be expressed as:

(A4) $${\text{v}}_{\left(k+1\right)}+\delta {\text{v}}_{\left(k+1\right)}={\text{v}}_{k}+\delta {\text{v}}_{k}+(I_{3\times 3}+{\left[K\right]}_{x}){\text{R}}_{k}^{⊤}\text{g}\Delta t-{\text{a}}_{m}\Delta t+{\text{b}}_{k}\Delta t+\delta {\text{b}}_{k}\Delta t$$

Segregating the error terms from both sides of Equation (A4), we obtain the expression to predict the error in the linear velocity:

(A5) $$\delta {\text{v}}_{\left(k+1\right)}=\delta {\text{v}}_{k}+{\left[K\right]}_{x}{\text{R}}_{k}^{⊤}\text{g}\Delta t+\delta {\text{b}}_{k}\Delta t=\delta {\text{v}}_{k}+{\left[K\right]}_{x}{\text{g}}_{L}\Delta t+\delta {\text{b}}_{k}\Delta t$$

Since [*K*]*_x_* contains the vector corresponding to the rotation error $\delta {\text{R}}_{k}^{⊤}$ and the acceleration bias is expressed as *δ***a***_k_* in the error state vector, Equation (A5) can also be seen as:

(A6) $$\delta {\text{v}}_{\left(k+1\right)}=\delta {\text{v}}_{k}-({\text{g}}_{L}⊗\delta {\text{q}}_{k})\Delta t+\delta {\text{a}}_{k}\Delta t$$

### B. Position Error Prediction

Let **p̃***_k_* be the estimated vehicle position defined according to Equation (23) as:

(A7) $${\tilde{\text{p}}}_{\left(k+1\right)}={\tilde{\text{p}}}_{k}+{\text{R}}_{k}{\tilde{\text{v}}}_{k}\Delta t+\frac{1}{2}{\text{R}}_{k}{\tilde{\text{a}}}_{k}\Delta t^{2}$$

According to Equations (1) and (A7), it can be expressed as:

(A8) $${\text{p}}_{\left(k+1\right)}+\delta {\text{p}}_{\left(k+1\right)}={\text{p}}_{k}+\delta {\text{p}}_{k}+{\text{R}}_{k}\delta {\text{R}}_{k}({\text{v}}_{k}+\delta {\text{v}}_{k})\Delta t+\frac{1}{2}{\text{R}}_{k}\delta {\text{R}}_{k}({\text{a}}_{k}+\delta {\text{a}}_{k})\Delta t^{2}$$

Analogously to Equation (A4), the term *δ* *_k_* is substituted by its approximation computed from the Rodrigues formula:

(A9) $${\text{p}}_{\left(k+1\right)}+\delta {\text{p}}_{\left(k+1\right)}={\text{p}}_{k}+\delta {\text{p}}_{k}+{\text{R}}_{k}(I_{3\times 3}+{\left[K\right]}_{x})({\text{v}}_{k}+\delta {\text{v}}_{k})\Delta t+\frac{1}{2}{\text{R}}_{k}(I_{3\times 3}+{\left[K\right]}_{x})({\text{a}}_{k}+\delta {\text{a}}_{k})\Delta t^{2}$$

Operating Equation (A9) and separating the nominal and the error terms in both sides of the expression, it gives:

(A10) $${\text{p}}_{\left(k+1\right)}={\text{p}}_{k}+{\text{R}}_{k}{\text{v}}_{k}\Delta t+\frac{1}{2}{\text{R}}_{k}{\text{a}}_{k}\Delta t^{2}$$

for the nominal position, and

(A11) $$\delta {\text{p}}_{\left(k+1\right)}=\delta {\text{p}}_{k}+{\text{R}}_{k}\delta {\text{v}}_{k}\Delta t+{\text{R}}_{k}{\left[K\right]}_{x}{\text{v}}_{k}\Delta t+{\text{R}}_{k}{\left[K\right]}_{x}\delta {\text{v}}_{k}\Delta t+\frac{1}{2}{\text{R}}_{k}\delta {\text{a}}_{k}\Delta t^{2}+\frac{1}{2}{\text{R}}_{k}{\left[K\right]}_{x}{\text{a}}_{k}\Delta t^{2}+\frac{1}{2}{\text{R}}_{k}{\left[K\right]}_{x}\delta {\text{a}}_{k}\Delta t^{2}$$

for the error in position.

Assuming that errors are very small between two filter iterations, taking into account that [*K*]*_x_* contains the rotation vector error and representing any product by [*K*]*_x_* as a cross product, Equation (A12) can be re-formulated as:

(A12) $$\delta {\text{p}}_{\left(k+1\right)}=\delta {\text{p}}_{k}+{\text{R}}_{k}\delta {\text{v}}_{k}\Delta t-{\text{R}}_{k}({\text{v}}_{k}⊗\delta {\text{q}}_{k})\Delta t+\frac{1}{2}{\text{R}}_{k}\delta {\text{a}}_{k}\Delta t^{2}-\frac{1}{2}{\text{R}}_{k}({\text{a}}_{k}⊗\delta {\text{q}}_{k})\Delta t^{2}$$

## References

[1] Smith S.M., An P.E., Holappa K., Whitney J., Burns A., Nelson K., Heatzig E., Kempfe O., Kronen D., Pantelakis T. (2001). The Morpheus ultramodular autonomous underwater vehicle. IEEE J. Ocean. Eng..

[2] Watson S.A., Crutchley D., Green P. (2012). The mechatronic design of a micro-autonomous underwater vehicle. J. Mechatron. Autom..

[3] Wick C., Stilwell D. A miniature low-cost autonomous underwater vehicle.

[4] Heriot Watt University mAUV. http://www.osl.eps.hw.ac.uk/virtualPages/experimentalCapabilities/Micro%20AUV.php.

[5] Hildebrandt M., Gaudig C., Christensen L., Natarajan S., Paranhos P., Albiez J. Two years of experiments with the AUV Dagon—A versatile vehicle for high precision visual mapping and algorithm evaluation.

[6] Kongsberg Maritime REMUS. http://www.km.kongsberg.com/ks/web/nokbg0240.nsf/AllWeb/D241A2C835DF40B0C12574AB003EA6AB?OpenDocument.

[7] Carreras M., Candela C., Ribas D., Mallios A., Magi L., Vidal E., Palomeras N., Ridao P. SPARUS. CIRS: Underwater, Vision and Robotics. http://www.cirs.udg.edu/auvs-technology/auvs/sparus-ii-auv/.

[8] Wang B., Su Y., Wan L., Li Y. Modeling and motion control system research of a mini underwater vehicle.

[9] Yu X., Su Y. Hydrodynamic performance calculation of mini-AUV in uneven flow field.

[10] Liang X., Pang Y., Wang B. (2009). Chapter 28, Dynamic modelling and motion control for underwater vehicles with fins. Underwater Vehicles.

[11] Roumeliotis S., Sukhatme G., Bekey G. Circumventing dynamic modeling: Evaluation of the error-state Kalman filter applied to mobile robot localization.

[12] Allotta B., Pugi L., Bartolini F., Ridolfi A., Costanzi R., Monni N., Gelli J. (2014). Preliminary design and fast prototyping of an autonomous underwater vehicle propulsion system. Inst. Mech. Eng. Part M: J. Eng. Marit. Environ..

[13] Kelly J., Sukhatme G. (2011). Visual-inertial sensor fusion: Localization, mapping and sensor-to-sensor self-calibration. Int. J. Robot. Res..

[14] Allotta B., Pugi L., Costanzi R., Vettori G. Localization algorithm for a fleet of three AUVs by INS, DVL and range measurements.

[15] Allotta B., Costanzi R., Meli E., Pugi L., Ridolfi A., Vettori G. (2014). Cooperative localization of a team of AUVs by a tetrahedral configuration. Robot. Auton. Syst..

[16] Higgins W. (1975). A comparison of complementary and Kalman filtering. IEEE Trans. Aerosp. Electron. Syst..

[17] Chen S. (2012). Kalman filter for robot vision: A survey. IEEE Trans. Ind. Electron..

[18] An E. A comparison of AUV navigation performance: A system approach.

[19] Suh Y.S. (2010). Orientation estimation using a quaternion-based indirect Kalman filter With adaptive estimation of external acceleration. IEEE Trans. Instrum. Meas..

[20] Miller P.A., Farrell J.A., Zhao Y., Djapic V. (2010). Autonomous underwater vehicle navigation. IEEE J. Ocean. Eng..

[21] Achtelik M., Achtelik M., Weiss S., Siegwart R. Onboard IMU and monocular vision based control for MAVs in unknown in- and outdoor environments.

[22] Weiss S., Achtelik M., Chli M., Siegwart R. Versatile distributed pose estimation and sensor self calibration for an autonomous MAV.

[23] Markley F.L. (2003). Attitude error representations for Kalman filtering. J. Guid. Control Dyn..

[24] Hall J.K., Knoebel N.B., McLain T.W. Quaternion attitude estimation for miniature air vehicles using a multiplicative extended Kalman filter.

[25] Quigley M., Conley K., Gerkey B., Faust J., Foote T., Leibs J., Wheeler R., Ng A. ROS: An open source robot operating system.

[26] Negre P.L., Bonin-Font F. GitHub. https://www.github.com/srv/pose_twist_meskf_ros.

[27] Hogue A., German J.Z., Jenkin M. Underwater 3D mapping: Experiences and lessons learned.

[28] MEMSENSE nIMU Datasheet. http://www.memsense.com/docs/nIMU/nIMU_Data_Sheet_DOC00260_RF.pdf.

[29] Fraundorfer F., Scaramuzza D. (2012). Visual odometry. Part II: Matching, robustness, optimization and applications. IEEE Robot. Autom. Mag..

[30] Geiger A., Ziegler J., Stiller C. StereoScan: Dense 3D reconstruction in real-time.

[31] Wirth S., Negre P., Oliver G. Visual odometry for autonomous underwater vehicles.

[32] Gracias N., Ridao P., Garcia R., Escartin J., L'Hour M., C. F., Campos R., Carreras M., Ribas D., Palomeras N., Magi L. Mapping the Moon: Using a lightweight AUV to survey the site of the 17th Century ship “La Lune”.

[33] Hartley R., Zisserman A. (2003). Multiple View Geometry in Computer Vision.

[34] Fossen T. (1994). Guidance and Control of Ocean Vehicles.

[35] Trawny N., Roumeliotis S.I. (2005). Indirect Kalman filter for 3D attitude estimation.

[36] Bonin-Font F., Beltran J., Oliver G. Multisensor aided inertial navigation in 6DOF AUVs using a multiplicative error state Kalman filter.

[37] Miller K., Leskiw D. (1987). An Introduction to Kalman Filtering with Applications.

[38] Ahmadi M., Khayatian A., Karimaghaee P. Orientation estimation by error-state extended Kalman filter in quaternion vector space.

[39] Bujnak M., Kukelova S., Pajdla T. (2011). New efficient solution to the absolute pose problem for camera with unknown focal length and radial distortion. Lect. Notes Comput. Sci..

[40] Fischler M., Bolles R. (1981). Random sample consensus: A paradigm for model fitting with applications to image analysis and automated cartography. Commun. ACM.

[41] Moore T., Purvis M. Charles River Analytics. http://wiki.ros.org/robot_localization.

[42] Bonin-Font F. Youtube. http://www.youtu.be/kKe1VzViyY8.

[43] Negre P.L., Bonin-Font F., Oliver G. Stereo graph SLAM for autonomous underwater vehicles.

[44] Ude A. (1999). Filtering in a unit quaternion space for model-based object tracking. Robot. Auton. Syst..

